# A new transdisciplinary research model to investigate and improve the health of the public

**DOI:** 10.1093/heapro/daaa125

**Published:** 2021-01-15

**Authors:** Helen Pineo, Eleanor R Turnbull, Michael Davies, Mike Rowson, Andrew C Hayward, Graham Hart, Anne M Johnson, Robert W Aldridge

**Affiliations:** 1 Institute for Environmental Design and Engineering, Bartlett School of Environment, Energy and Resources, University College London, Central House, 14 Upper Woburn Place, London WC1H 0NN, UK; 2 Academic Foundation Programme, North Central and East London Foundation School, Health Education England, Stewart House, 32 Russell Square, Bloomsbury, London, UK; 3 Centre for Public Health Data Science, Institute of Health Informatics, University College London, 222 Euston Road, London, NW1 2DA, UK; 4 Faculty of Population Health Sciences, University College London, 149 Tottenham Court Road, London, W1T 7BN, UK; 5 Institute of Epidemiology and Health Care, University College London, 1-19 Torrington Place, London, WC1E 7HB, UK; 6 Institute for Global Health, University College London, Mortimer Market Centre, Capper Street, London, WC1E 6JB, UK

**Keywords:** transdisciplinary, public health, methodology, research process

## Abstract

Transdisciplinary research approaches are being applied to today’s complex health problems, including the climate crisis and widening inequalities. Diverse forms of disciplinary and experiential knowledge are required to understand these challenges and develop workable solutions. We aimed to create an updated model reflective of the strengths and challenges of current transdisciplinary health research that can be a guide for future studies. We searched Medline using terms related to transdisciplinary, health and research. We coded data deductively and inductively using thematic analysis to develop a preliminary model of transdisciplinary research. The model was tested and improved through: (i) a workshop with 27 participants at an international conference in Xiamen, China and (ii) online questionnaire feedback from included study authors. Our revised model recommends the following approach: (i) *co-learning*, an ongoing phase that recognizes the distributed nature of knowledge generation and learning across partners; (ii) *(pre-)development*, activities that occur before and during project initiation to establish a shared mission and ways of working; (iii) *reflection and refinement* to evaluate and improve processes and results, responding to emergent information and priorities as an ongoing phase; (iv) *conceptualization* to develop goals and the study approach by combining diverse knowledge; (v) *investigation* to conduct the research; (vi) *implementation* to use new knowledge to solve societal problems. The model includes linear and cyclical processes that may cycle back to project development. Our new model will support transdisciplinary research teams and their partners by detailing the necessary ingredients to conduct such research and achieve health impact.

## INTRODUCTION

Improving the health of the public in the 21st century requires actors from many sectors to co-produce knowledge and policy to solve complex global challenges affecting health (Kickbusch and Gleicher, 2012; The Academy of Medical Sciences, 2016; de Leeuw, 2017). Rapid urbanization, widening inequalities, climate change and the rising burden of chronic disease are all complex societal problems that affect health, but will not be solved by health researchers or practitioners working alone. These challenges are intractable partly due to their complexity. Transdisciplinarity is positioned as essential to understanding and finding solutions for global challenges by enabling a holistic view, integrating diverse knowledge and transcending disciplinary approaches (Nicolescu, 2002). Against the sense of powerlessness invoked by threats of the climate emergency and widening inequalities, transdisciplinarity offers hope that impactful solutions can be identified and implemented. 

Definitions and conceptualizations of integrative research vary widely in health research and other fields, and terms such as multi-, inter- and transdisciplinary are sometimes used interchangeably (Choi and Pak, 2006; Lynch, 2006; Stokols *et al.*, 2013). Transdisciplinary research is seen as the most integrative form of cross-disciplinary research (Czajkowski *et al.*, 2016) that was first created to solve complex problems in environmental sustainability (Berger-González *et al.*, 2016) and is used in environment and health research, such as EcoHealth approaches (Buse *et al.*, 2018).

There is consensus that transdisciplinary approaches involve integrating and transcending individual disciplines enabling development and application of new research strategies and knowledge, as set out by Rosenfield (Rosenfield, 1992). There is debate about whether such research is always conducted in teams (Choi and Pak, 2006; Tress *et al.*, 2006), whether it can be practiced individually or whether non-academic partners must be involved (Stokols *et al.*, 2013). Transdisciplinary research has been compared to community-based participatory research (CBPR) (Berger-González *et al.*, 2016) and translational science (Lynch, 2006), and we argue it overlaps with co-production (Oliver *et al.*, 2019). We adopt Stokols *et al.*’s definition of transdisciplinary research as ‘an integrative process whereby scholars and practitioners from both academic disciplines and non-academic fields work jointly to develop and use novel conceptual and methodological approaches that synthesize and extend discipline-specific perspectives, theories, methods, and translational strategies to yield innovative solutions to particular scientific and societal problems’ [(Stokols *et al.*, 2013), p. 6]. We recognize that other conceptualizations of transdisciplinarity co-exist and will be reviewed in our study. We also build upon their cyclical transdisciplinary framework, comprising development, conceptualization, implementation and translation phases (Hall *et al.*, 2012; Stokols *et al.*, 2013), as summarized in the Supplementary material.

A number of challenges are inherent to current transdisciplinary research practice. Foremost, research partners (be they academic or otherwise) may be driven by different assumptions or epistemological positions, creating tensions when conceptualizing approaches. Further challenges include: longer project duration, difficulty publishing in high impact journals or as a single author, challenges obtaining adequate research funding for larger and longer projects, falling between funding body remits, problems communicating within and beyond the team, researcher vulnerability to adverse psychological impacts, and avoiding social pressures to implement research with a single focus (Lynch, 2006; Lang *et al.*, 2012; Davies *et al.*, 2015; Hébert *et al.*, 2016; Black *et al.*, 2019). Despite these issues, Abrams argues that ‘scientists who succeed in embracing a transdisciplinary approach experience it as a tipping point in their career, enhancing their professional growth and creativity’ [(Abrams, 2006), p. 516].

In summary, our research responds to the great potential of transdisciplinarity, whilst recognizing its challenges, by developing an updated transdisciplinary model appropriate for investigating and improving health and its wider determinants and building capacity to adopt transdisciplinary approaches. By ‘model’ we mean a simplified representation of how something works (Johnson-Laird, 1983). Although there is no ‘correct’ way to design a transdisciplinary research project (Ciesielski *et al.*, 2017), we perceive Stokols *et al.*’s (Stokols *et al.*, 2013) cyclical process to be insufficiently descriptive of our experience and the literature. Our research extends their framework by reflecting lessons learned from practice and therefore serves dual purposes for training new ‘transdisciplinarians’ and enabling further improvement to research practice, specifically related to health and its wider determinants. Our aim is to support trandisciplinary researchers with an updated model that fully captures its potential and reality to inform new research strategies and reporting.

## METHODS

We conducted a literature review using OVID Medline (27 May 2019) exploring articles that conducted transdisciplinary research. We conducted a narrative synthesis using thematic analysis, coding studies inductively and deductively at the semantic level, using an a priori codebook for deductive coding using Stokols *et al.*’s (Stokols *et al.*, 2013) transdisciplinary research framework. We adopted their four phases as coding categories (development, conceptualization, implementation and translation) and we created codes within these using their goals and processes. We developed a new preliminary model of transdisciplinary research and gathered feedback from experts in transdisciplinary research by contacting the included study authors using email and an online Google form (RWA) and by conducting a participatory workshop with 27 participants (HP, MD). The workshop was held at the 16th International Conference on Urban Health in Xiamen, China on 4 November 2019 at a pre-conference session co-organized by the Wellcome Trust and their funded research projects under the Our Planet Our Health (OPOH) programme (Pineo *et al.*, 2020). Workshop participants (WPs) received a presentation with Stokols *et al.*’s (Stokols *et al.*, 2013) definition and model of transdisciplinary research before introducing the research methods and preliminary model. We describe our methods more fully in the Supplementary material including: search terms, data extraction, coding and synthesis approach, and online questionnaire and workshop details.

## OVERVIEW OF INCLUDED STUDIES AND EXPERT FEEDBACK

Our search provided 529 results and following screening, 29 studies met our inclusion criteria (see Supplementary material for the flow of results and summary of included studies). The included studies aimed to study and/or directly influence a diverse range of health conditions and wider determinants of health. There were 93 unique fields/disciplines (from a total of 157 reported across studies) represented across the following classifications: Health and Life Sciences (67%, 105/157), Economic and Social (15%, 23/157), Engineering and Physical Sciences (12%, 19/157) and Arts and Humanities (6%, 10/157). Many participants at our workshop identified themselves as experienced in transdisciplinary research with 78% (21/27) having used such approaches. WPs and online questionnaire respondents (QRs) suggested changes or agreed with our model and we used their views to reach the version presented here (see Supplementary material for details). In presenting our model, we describe findings from included studies, WPs and QRs.

## A NEW MODEL FOR TRANSDISCIPLINARY RESEARCH

**Fig. 1:**
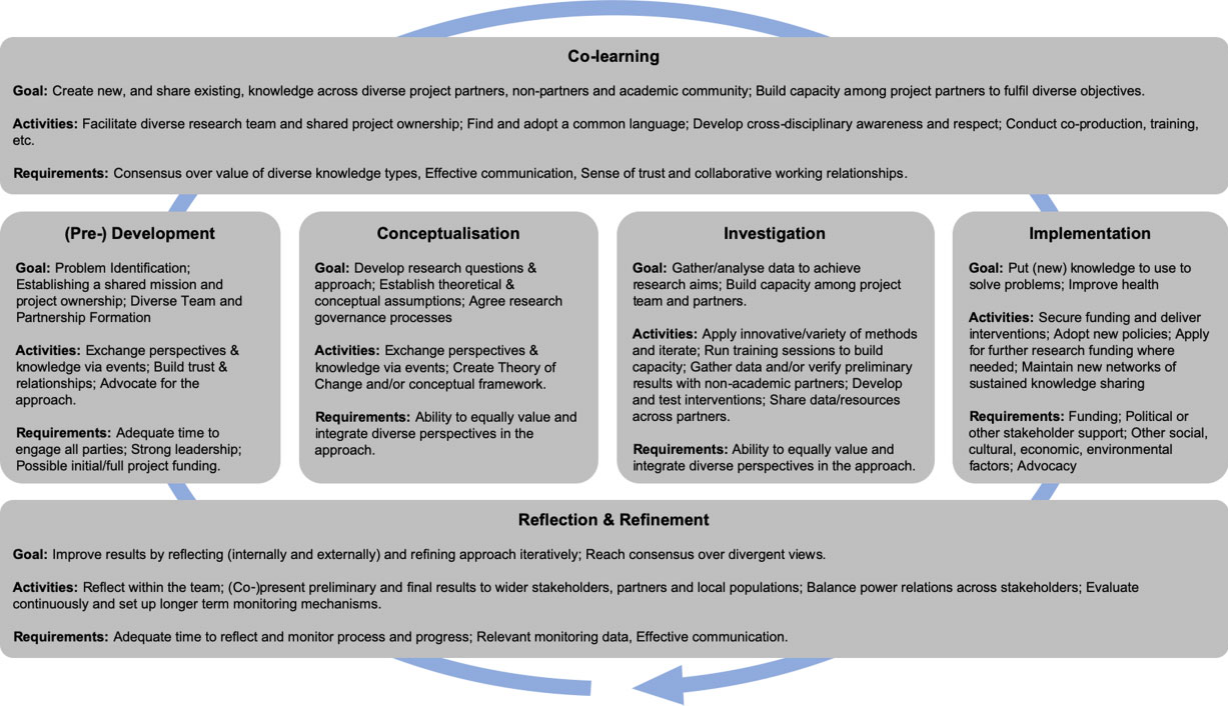
New model of transdisciplinary research (detailed version).

Our proposed model (Figure 1) contains elements of the Stokols *et al.*’s (Stokols *et al.*, 2013) framework. However, there are key changes (outlined below). Our updated model (Figure 1) provides an explanation of goals, activities and requirements at each phase. The Supplementary material contains a simplified version of the model for the purposes of communicating the overall process and relations between phases with wide stakeholders. Our new model emphasizes the interconnections between phases and iterative nature of transdisciplinary research and as a result includes linear and cyclical processes that may feedback to project development. Our revised model has six main phases: (i) *co-learning*, a cross-cutting theme that recognizes the distributed nature of knowledge generation and learning across partners; (ii) *(pre-)development* to demonstrate the wide range of activities that occur prior to (and during) official project initiation; (iii) *reflection and refinement* to evaluate and improve processes and results, responding to emergent information and priorities as an ongoing process; (iv) *conceptualization* to develop research questions and the study approach (etc.) by combining diverse knowledge; (v) *investigation* to conduct the transdisciplinary research; (vi) *implementation* to put new knowledge to use to solve problems.

In comparison with Stokols *et al.*’s (Stokols *et al.*, 2013) framework, we have made six key changes. We replaced their ‘Translation’ phase with a new cross-cutting ‘co-learning’ phase. We added the ‘investigation’ phase in which research is conducted and re-purposed Stokols *et al.*’s ‘implementation’ phase so that it no longer refers to doing research and is now about making change happen beyond the research process. We added the ‘reflection and refinement’ phase as a cross-cutting process that recognizes the need for ongoing evaluation and iteration over time. We have drawn out the pre-development activities in Stokols *et al.*’s ‘Development’ phase to demonstrate the wide range of time-consuming activities that occur prior to official project initiation. Finally, we modified the flow of the diagram from a cycle to include linear and cyclical processes that may feedback to project development, but not necessarily.

### Co-learning

In this cross-cutting phase knowledge is created and shared continuously with a diverse range of project partners and wider audiences, in stark contrast to traditional dissemination models that publish findings at the end of a study. Learning occurs throughout the research process, not only through traditional project deliverables. For example, Harper *et al.* worked with the Rigolet Inuit community to understand their experiences through storytelling to co-develop ‘culturally appropriate climate-related health policies and programs that reflect Indigenous perspectives and values’ [(Harper *et al.*, 2012), p. 94]. The authors reported that this collaboration ‘between researchers, community members, and decision makers […] allowed the integration of knowledge and synthesized new theories, concepts, and methods that would not have otherwise emerged’ (Harper *et al.*, 2012). The learning is not only by researchers but also occurs with communities and wider stakeholders who identify new approaches and ideas. Ramey *et al.* explained: ‘we have difficulty accurately identifying which ideas and novel features ‘‘came from’’ the community versus academic partners, because the close collaborations we developed supported a sense of shared innovation and insight’ [(Ramey *et al.*, 2015), p. e5]. In addition, a WP wrote ‘For [the project goal] to happen we have to equally include everyone, and learn from one another’. Building capacity across project partners was a key goal of this phase, and referred to ‘skills, knowledge and empowerment’ [(Mutero *et al.*, 2004), p. 183] which was sometimes a result of the research or in other cases was actively pursued through training or awareness campaigns.

One activity that enables co-learning and is fundamental to transdisciplinary research is pursuing a common language across diverse partner backgrounds (and indeed formal languages, where relevant). This was highlighted by study authors and QRs as challenging and necessary, e.g. ‘technical terms that are self-evident in one discipline might be understood differently or unknown in others’ (QR). This activity could be achieved through encouraging researchers to explain terms (QR) or through adopting a method that transcends language such as participatory photo mapping (Dennis *et al.*, 2009). Developing a shared language was recognized as an ongoing process that requires continual checking for understanding as research methods, analyses and results are discussed.

Developing a shared language is not only about avoiding misunderstanding of technical terms but also relates to valuing diverse perspectives, shown by the ‘Develop cross-disciplinary awareness and respect’ activity (Figure 1). One QR said: ‘a computational scientist is looked down upon when she attempts to discuss social science with an expert in this area’. Lytle underscored the importance of developing respect for diverse epistemologies through her definition of transdisciplinary research, stating that it ‘involves cross-fertilization between disciplines that includes finding a shared language, building tolerance for looking at an issue through a completely different lens, and using creativity to use new and old models, measures and analytic methods to solve complex problems’ [(Lytle, 2009), p. 339]. Good communication and consensus building across disciplines/stakeholders to develop such tolerance and respect could be facilitated by a knowledge broker (Fam and Sofoulis, 2017) or carrying out a team ‘orientation exercise’ (QR) to expose and work through issues. The ability to equally value and integrate diverse perspectives in the approach needs to be actively fostered throughout the research (examples are given in the following sections).

A final key requirement for co-learning is maintaining trust within the project team and with wider stakeholders, such as local populations, which is also described as an ongoing process. The latter is particularly important when people have previous negative experiences with researchers or about the study topic, e.g. where local populations have been stigmatized, stereotyped, analyzed and exploited (Harper *et al.*, 2012; Anticona *et al.*, 2013; Ziegler *et al.*, 2016).

### (Pre-)development

The transdisciplinary approach starts before and overlaps with the processes of problem definition, identification of the research question and design of the research strategy (the ‘conceptualization’ process), when a diverse range of partners meet to discuss a mutually agreed problem. At the ‘(pre-)development’ phase stakeholders establish shared project ownership and a ‘shared mission’ (WP) before forming a partnership, potentially to seek funding. Researchers recognized that diverse project participation was essential: ‘revitalis[ing] informal settlements… cannot be done one-directionally. And it requires different stakeholders (not just academic) to come together and work together’ (WP)*.* Having the resources and interest to devote time to pre-development and other early project activities may not be equally shared across all partners. One WP highlighted the importance of identifying possibilities for sharing resources because it ‘decides what kind of results could be achieved’. This could be in relation to financial or other resources (such as project management support or data). Projects used events and meetings to exchange perspectives and knowledge about the topic, to build relationships and trust among stakeholders and to advocate for funding or other resources. Identification of the research problem, questions and strategy were described as ongoing and subject to change.

The processes of team and project development require significant time to engage all parties, agree upon and progress the shared mission, and identify or secure appropriate resources. Several studies reported setting up advisory (or steering) committees to provide scientific oversight with external members (Ramey *et al.*, 2015) or internal committees to run the study (shown under ‘conceptualization’ in Figure 1). For instance, Anticona *et al.*’s ‘operational research committee’ (ORC) was comprised of representatives from all research partners and ensured that they could ‘contribute equally and share control of the research process’ [(Anticona *et al.*, 2013), p. 3)]. However, the authors reflected that the members of the ORC had ‘limited power and a low degree of authority’ thus creating delays in decision-making (Anticona *et al.*, 2013). This resulted in project leaders going around the ORC to get quicker decisions, creating conflict as team members felt side-lined. The steering committee in Ramey *et al.* put in place a voting system to avoid power imbalances between academic and ‘community’ representatives in which ‘each site or entity in the network would have a single vote, thus further strengthening local partnerships to work efficiently, discover their own unified voice, and increase respect for and knowledge about diverse perspectives’ [(Ramey *et al.*, 2015), p. 3]. Although these research governance mechanisms were discussed, many studies did not provide information about how research teams were formed at the beginning of projects.

Key requirements for success at this early phase, and throughout the project, relate to characteristics of team members, including strong leadership, communication and team-working skills that support development of project governance structures and overall success of the project. Regarding leadership, Black and Black explained ‘those leaders who excel at generating and sustaining trust, who are supportive, democratic, inclusive, empowering, and are committed to encouraging cooperation and engaging the support of others by being generous in offering constructive feedback to colleagues will significantly enhance trans-disciplinary collaborations within the research team, within the research institute or university and amongst key stakeholders’ [(Black and Black, 2009), p. 1582]. A study author provided a word of caution about the role of leadership by noting that if a project leader pursues a disciplinary approach this can derail transdisciplinary ambitions, instead this person should focus on ‘bringing people together and insisting on finding common solutions/consensus’ (QR).

### Reflection and refinement

Reflection and refinement, including monitoring and evaluation, occurs continually as well as at specific points in a project. Continual reflection and adaptation of the project (including the research questions, partners, conceptual framework and research strategy) is required as new information and priorities arise. Specific points of reflection include checking initial results (optionally by co-presenting results) with relevant stakeholders to: validate or improve findings, prioritize results; gain further understanding of results in the local context; and raise and discuss any discrepancies. For example, Ingemann *et al.* (Ingemann *et al.*, 2018) report their data collection strategy in a flow diagram whereby ‘reflect’ and ‘plan’ arrows connect each of the six data collection phases, demonstrating the iterative and reflective nature of their project. Following refinements, a further dissemination and validation meeting with the stakeholders can: support collective decision-making about what to address next, uncover solutions for how to tackle a problem, reach consensus over diverse views and increase legitimacy and ownership for the next phase of research.

This ongoing phase requires teams to recognize and act upon problems that emerge in the project such as power imbalances, conflict between partners and leadership changes in academic or partner organizations. Berger-González *et al.* applied a multi-staged process ‘to “equalize” power differentials inherent in the knowledge systems under study’ [(Berger-González *et al.*, 2016), p. 81]. For instance, this involved joint chairs for the project’s opening meeting (one Mayan elder and one senior academic), sharing participants’ expectations openly (which were collated and reported in the manuscript (Berger-González *et al.*, 2016), reflecting diverse interests among the project partners) and agreeing the final study protocol and interview guide through intensive sessions lasting 2 and 3 days, respectively. Artistic methodologies were suggested by a WP to help make ‘power relations more explicit/emergent’. For example, Harper *et al.* explained that by using digital storytelling the participants, not the researchers, decide which data are important, thus reversing the typical power structures in research (Harper *et al.*, 2012).

Monitoring and evaluation objectives span the day-to-day running of the project and efforts to measure the medium- to long-term impact of the project on wider outcomes. Evaluation was not widely described in the included studies, although WPs highlighted the importance of setting up monitoring and evaluation mechanisms, requiring available data. With regard to evaluation of the transdisciplinary process itself, Holmes *et al.* found that self-reported data about collaboration were not adequate to map the ‘evolution of team science’, instead they advocated new approaches including ‘temporal social-network analysis and a formal bibliometric analysis of not only published but cited publications as ways to investigate the emergence of “new science”’ [(Holmes *et al.*, 2008), p. S191].

### Conceptualization

The purpose of the ‘conceptualization’ phase is to ensure development of comprehensive questions and appropriate methods to uncover innovative solutions to complex health problems. In Stokols *et al.*’s (Stokols *et al.*, 2013) model, research questions and strategies are informed by a shared conceptual framework that incorporates theoretical and conceptual assumptions. WPs identified the impracticality of this collaborative approach because funding may be secured by investigators who have pre-defined the strategy before all partners are on-board. The ‘conceptualization’ process (Figure 1) is still early in the project timeline and involves foundation-setting activities such as agreeing research governance processes [overlapping with ‘(pre-)development’] and developing the research approach. As in the ‘(pre-)development’ phase, teams used events and meetings to establish ‘common understanding of the issue’s complex nature and for coherently framing research objectives’ [(Ziegler *et al.*, 2016), p. 319].

Studies described various processes for developing a conceptual framework, often beginning with a literature review and using participatory approaches to facilitate agreement of a shared vision. The process provides an opportunity to explore the complex relations between risk factors and intervention opportunities and ‘identify testable hypotheses about divergent and co-existing pathways to better (vs. compromised) health’ [(Ramey *et al.*, 2015), p. e4]. An incomplete understanding of such linkages may lead to inappropriate analyses and reduce the effectiveness of any future interventions. An initial conceptual framework should be further validated and adapted by all relevant academic and non-academic stakeholders, therefore linking to the ‘reflection and refinement’ phase. This can be done through multiple methods (including community meetings, focus groups, and interviews) ensuring the involvement and ownership of stakeholders with diverse perspectives, a key requirement of this phase.

### Investigation

As in previous phases, the ‘investigation’ phase includes active involvement across partners to collect and analyse data, with continuous reflection and refinement leading to co-learning. Data collection methods are varied (see Supplementary material) and include the production of innovative methods. Many projects used mixed methods approaches, enabling triangulation of data informed by different disciplines, although some projects were exclusively qualitative or quantitative in nature. To integrate disciplinary and practitioner knowledge in data analysis, Quintero *et al.* ‘applied a multiple triangulation analysis by combining theoretical and methodological triangulations’ whereby multiple perspectives (from psychology, medicine, anthropology, etc.) were involved in interpreting the data [(Quintero *et al.*, 2009), p. S95]. The ‘investigation’ process may involve the development and/or testing of specific interventions that may later be implemented more widely.

WPs discussed a range of challenges with data collection and analysis, including the balance between these traditional research activities and other more time-consuming collaboration and team management activities, such as attending internal and external meetings. This phase supports capacity building (part of ‘co-learning’) among project members and local populations who learn new knowledge and skills. Capacity building was supported by running training sessions on new data collection methods.

### Implementation 

The purpose of the ‘implementation’ phase is to put new knowledge to use to solve problems, improving health and its wider determinants. Studies used a range of activities in this phase to affect change, including: internal or external stakeholders used results to improve health or the wider determinants of health; the project developed, ran and evaluated an intervention; project partners sought additional funding, cycling back to the ‘(pre-)development’ phase; and partners used the research outputs for advocacy, education or increasing research in an under-represented topic. These examples are elaborated in the Supplementary material.

Depending on the field of inquiry (e.g. upstream determinants of health versus downstream healthcare service delivery) implementation activities may be more or less likely to be led by non-academic partners. As one WP wrote, ‘I’m not sure about the term “implementation.” Investigators rarely directly implement any policy levers/strategies – usually they inform, “translate”, “advocate”’. Similarly, participants and studies noted that this phase is not likely to be funded or controlled by the transdisciplinary project and will therefore require funding and political or other stakeholder support; and will be affected by social, cultural, economic, and environmental factors.

## REFLECTIONS FROM THE FIELD

In addition to the analysis above, we believe that some transdisciplinary challenges and opportunities could be further elaborated in support of our proposed model. Here, we reflect on our transdisciplinary experience to fill those gaps.

Achieving the aims of the ongoing ‘co-learning’ phase requires active involvement from diverse project partners, yet this depends on funding and other enablers to participation (such as personal interest, organizational incentives and more). In the Homeless Hospital Discharge study, people with experience of homelessness were project partners throughout and they had a strong interest (or stake) in the project. The study found high rates of emergency readmissions across all disease categories for people experiencing homelessness admitted to hospital (Lewer *et al.*, 2019). This finding was interpreted by one of the partners (and co-author) with experience of homelessness as relating to medical professionals overly focusing on such patients as ‘bed-blockers’ rather than fully treating their conditions [(Lewer *et al.*, 2019), p. 15]. This partner is now using knowledge gained about health and homelessness from this study in his new job running a homeless hostel. In other cases, we have encountered barriers to participation for some non-academic partners. For example, in the Complex Systems for Sustainability and Health (CUSSH) project (https://www.ucl.ac.uk/complex-urban-systems/), delays in establishing memoranda of understanding with local governments in several of the cities meant that it was difficult for officials, initially at least, to engage fully in the work. The reasons for the delays were not anticipated and were different in each case. Creative solutions were required which provided valuable learning for future work. 

The ‘(pre-)development’ phase of project formation deserves greater attention from both academics and funders. The early formation of inter-personal relationships across partners requires a facilitated and planned set of activities to work through the challenges to transdisciplinarity (e.g. power imbalances and finding a shared language). The UCL–*Lancet* Commission on Migration and Health (Abubakar *et al.*, 2018) involved a 3-day working session at a residential facility where Commissioners drafted sections of the paper and debated topics for inclusion. Team-building activities and working sessions helped: stimulate exchanges across disciplines; build rapport among and empower Commissioners at all career stages; and explore partners’ individual expectations from the project. This event did not fully resolve all project conflicts (e.g. challenges with conceptual framings and disciplinary language), however, it went a long way in establishing inter-personal relationships in which Commissioners could negotiate compromises and achieve consensus for the final report. This was important to overcome the difficulties associated with doing research across many countries and disciplines (including academic and international governmental organizations). In summary, co-learning and forming an effective team are not automatic outcomes of transdisciplinary studies; they require planning, funding and willingness among all partners to engage.

## RECOMMENDED REPORTING OF TRANSDISCIPLINARY RESEARCH

Gathering insights from the included studies has shown us the value of published case studies and reflections on the transdisciplinary research process. If more projects describe the complex, costly, time-consuming, yet potentially rewarding process of such research, other research teams may be in a better position to argue the case for funding and innovative practices/structures across institutions. We recommend that studies report the process-related factors in our recommended reporting criteria below (see Supplementary material for a table), as appropriate for the individual study. This need not be a tick-box reporting exercise, but instead we aim for this to serve as a useful catalyst for reflection during the project from which others can learn.

*(Pre-)development*: How was the project team formed? Which organizations are included and what are their roles? How did partners agree on the problem or mission?

*Co-learning*: Who attended project meetings? What activities were used to create or share knowledge with diverse stakeholders? If relevant, how were conflicts resolved about the value of diverse knowledge types (e.g. technical knowledge, experiential knowledge, etc.)? How did the project build capacity within and beyond project partners? How did partners build and maintain trust throughout the project?

*Reflection and refinement*: Which processes (or activities) were used to reflect upon the research and emerging results? Which indicators were used to monitor progress/impact? Who managed/participated in monitoring and reflection processes?

*Conceptualization*: How were diverse assumptions, theories and knowledge types integrated to form the project’s research questions and approach? Which research governance processes were established and were these adjusted over time? Did the project create/adopt a conceptual framework and/or Theory of Change and how did this occur? Which activities were used to exchange perspectives and knowledge when conceptualizing the research approach and who was involved?

*Investigation*: Which methods were used to gather and analyse data and how were these methods novel or integrative? How did the ‘investigation’ phase build capacity within and beyond project partners? Who was involved in gathering and analysing data and what were their respective roles?

*Implementation*: How was (new) knowledge from this project used to solve problems and or improve health? Or how do partners anticipate such change occurring? Which factors were required to make this change happen (e.g. funding or political will) and how were these achieved?

## DISCUSSION

Although it is difficult and costly, transdisciplinary research is increasingly regarded as an essential approach for investigating complex health problems (The Academy of Medical Sciences, 2016). Our new model aims to support transdisciplinary research teams by detailing the necessary ingredients to conduct such research, with the ambition of improving the health of the public. We propose that our model may be iteratively improved to reflect changing practice, which will be aided by studies reporting their experiences using the proposed criteria. We applied a rigorous review and coding methodology to conduct a narrative synthesis and develop our model, which we iteratively tested through a participatory workshop and online questionnaire. However, the review excluded grey literature, unpublished research, research not indexed in Medline, non-English language papers and studies that did not reference/explain their transdisciplinary approach. Our literature search used the term transdisciplinary and as a result will have excluded studies that may have been transdisciplinary in nature but did not describe themselves as such. Below we reflect on wider debates related to transdisciplinarity that inform the interpretation and value of this article.

In theory, transdisciplinarity responds to the demands of complex societal problems by recognizing that academic knowledge and single discipline approaches will not be sufficient to understand causes and solutions for these issues. In reality, it is unclear how well existing academic structures and norms support transdisciplinary practices so that diverse knowledge is fully integrated and helps transform societal problems. Our analysis has highlighted the ongoing challenge of integrating diverse knowledge and shown that whilst compromises and conclusions were reached in the reported studies, there were also considerable conflicts. In this section we aim to provoke further debate about what transdisciplinary approaches *should* and *do* mean through discussion of three key challenges that we believe are core to transdisciplinary research: (i) participation, (ii) knowledge integration and (iii) moving from knowledge to action.

Full participation of people with diverse knowledge is integral to transdisciplinary research, yet our findings showed that achieving equal participation is a significant challenge. The literature on CBPR highlights the necessity of sharing power and building trust among partners to achieve equal participation (Jagosh *et al.*, 2015). Israel *et al.*’s nine guiding principles of CBPR are equally relevant for transdisciplinary challenges, a selection of which include: facilitating an empowering process that addresses inequalities, promoting ‘co-learning and capacity-building among all partners’, and achieving ‘a balance between research and action’ [(Israel *et al.*’s, 2019), p. 274]. Oliver *et al.* advocate reflective processes given the potential risks of co-production (extending beyond CBPR) which can ‘cause conflict, consume resources and lead to misunderstandings’ [(Oliver *et al.*, 2019), p. 3]. Our model emphasizes the importance of ongoing reflection to manage potential problems and risks for project partners (such as power imbalances and conflict) and the need for increased clarity over the research process (via our reporting criteria). We think that the risks of participatory research and associated solutions need to be fully acknowledged by researchers, funders and academic institutions if transdisciplinary collaboration is to succeed in solving pressing health challenges.

A simultaneous strength and challenge of transdisciplinary research is its goal to bring together, integrate and transcend different knowledge types. The literature sometimes positions academics and research funders as separate from ‘real world’ problems and solutions (Ingemann *et al.*, 2018; Black *et al.*, 2019), which implies a lack of confidence in academics’ ability to lead transdisciplinary research. Positioning academic and ‘real world’ knowledge as separate and mutually incomprehensible has parallels with Kuhn’s (Kuhn, 1962) argument that scholarly disciplines operate in different worlds. In line with critiques of Kuhn [see (Phillips, 1987)] we think that academics can understand and operate within systems beyond their disciplines and institutions. Many scholars are deeply embedded in practice and experience in their respective fields, such as clinical academics, engineers and urban planners. In considering the diverse knowledge that might be integrated in transdisciplinary research, we borrow from the Pastille Consortium’s analysis of science’s role in policy that distinguished three knowledge types: ‘socially accredited’ (e.g. scientific and technical), ‘experiential’ (e.g. gained through doing certain types of work or being in a particular environment) and ‘process’ (e.g. understanding how to achieve effects in a particular system) [(Pastille Consortium, 2002), p. 70]. To effectively operate across institutional or sectoral boundaries we agree that transdisciplinarians need an appreciation of different types of knowledge and this should be integrated into educational curricula (Gombrich and Hogan, 2017). This training may support researchers with the ability to not only have ‘tolerance for looking at an issue through a completely different lens’ [(Lytle, 2009), p. 339] but to cultivate the co-existence of diverse lenses within one’s scientific approach, seeing the strengths and weaknesses of applying different perspectives in different circumstances—in other words being independently transdisciplinary.

Finally, we consider a key aim of transdisciplinary research whereby partners from multiple epistemological positions determine what types of knowledge are deemed suitable to inform action. Scholars have argued that not all knowledge claims can be treated equally when knowledge informs action, such as for energy policies (Rydin, 2007). Yet, Popay (Popay, 2018) cautions that there can be grave consequences when certain knowledge is disregarded, such as residents’ evidence about fire risk at Grenfell Tower. Traditional knowledge translation models are predominately linear and use evidence-based policy models that do not apply to many complex cross-sector challenges (de Leeuw, 2017). Turning to planning scholarship may offer some helpful approaches for transdisciplinarians.

Rydin argues that in deciding how to act upon various knowledge types, planners should evaluate them critically recognizing that they are all value-laden. She offers an approach for planners to test and recognize knowledge claims whereby they should: support voices that have less power to contribute, organize forums for evaluating knowledge claims that include procedures to resolve conflicting claims, decide whether a proposed knowledge claim represents a ‘causal model [that] is sufficiently robust for decision-making’ and ensure that normative knowledge claim debates occur in the ‘public sphere where a range of voices can be heard’ [(Rydin, 2007), p. 65]. This could be adapted for transdisciplinary research. As Rydin describes, planning theory has also advocated for planners to co-produce knowledge with affected communities about local problems and solutions in recognition of the contested and power-laden nature of planning issues. Collaborative approaches, such as Innes and Booher’s (Innes and Booher, 2010) DIAD (Diversity, Interdependence, Authentic Dialogue) theory of collaborative rationality, may also help planners to understand and respond to complex problems by eliciting and sharing distributed knowledge. Nevertheless, collaborative planning approaches have been critiqued due to over-reliance on planners’ skills to undertake these processes, risk of being subverted by powerful interests, and potential inability to achieve consensus due to conflict among stakeholders (Rydin, 2007). In summary we want to highlight that transdisciplinary researchers are not alone in grappling with the difficult challenge of deciding how to integrate and act upon diverse knowledge types. Many academics prefer to operate as policy-neutral scholars to avoid reputation damage and conflict (Oliver and Cairney, 2019), yet by determining which knowledge is represented in research they influence policy discourses and agendas. In transdisciplinary research academics should be *part of* a group that *together* must decide how to integrate and act upon diverse knowledge types.

In conclusion, we have presented a new model for transdisciplinary research that has six phases of co-learning, (pre-)development, reflection and refinement, conceptualization, investigation and implementation. Our model occupies a middle position between being prescriptive (as it is based on other normative models) and being descriptive of practice. Further reflection and research on the process and outcomes of transdisciplinary research would help to establish its benefits and risks. Finally, we note the importance of training the next generation of transdisciplinarians and we advocate recognition of the specific skills and attitudes required for this approach in educational and professional accreditations.

## SUPPLEMENTARY MATERIAL

Supplementary material is available at *Health Promotion International* online.

## Supplementary Material

daaa125_Supplementary_Data
