# Supplementary material for: A new transdisciplinary research model to investigate and improve the health of the public
Source: Health Promot Int. 2021 Jan 15;36(2):481–92. doi: 10.1093/heapro/daaa125 (PMC8049543; doi:10.1093/heapro/daaa125)
Supplement: daaa125_Supplementary_Data [file daaa125_supplementary_data.docx]

Supplementary Material for ‘A new transdisciplinary research model to investigate and improve the health of the public’

Helen Pineo; Eleanor R. Turnbull; Michael Davies; Mike Rowson; Andrew C Hayward; Graham Hart; Anne M Johnson**;** Robert W. Aldridge

Table of Contents

[1. Definitions of integrative research 1](#_Toc54098021)

[2. Methods 2](#_Toc54098022)

[2.1. Workshop feedback form questions 3](#_Toc54098023)

[2.2. Preliminary model 3](#_Toc54098024)

[2.3. Online questionnaire questions 5](#_Toc54098025)

[3. Results 5](#_Toc54098026)

[3.1. Description of included studies 5](#_Toc54098027)

[3.2. Description of feedback and changes to the model 12](#_Toc54098028)

[3.3. Simplified version of the model 14](#_Toc54098029)

[3.4. Comparison with Stokols et al.’s framework 14](#_Toc54098030)

[3.5. Examples of implementation activities 14](#_Toc54098031)

[4. Recommended reporting of transdisciplinary research 15](#_Toc54098032)

[5. References 16](#_Toc54098033)

# Definitions of integrative research

Table 1 describes definitions of single discipline and integrative research from the literature. Table 2 summarises Stokols et al.’s (2013, p.9) four phases of transdisciplinary research, including goals and processes at each phase.

Table 1 Different forms of non-integrative and integrative research and their definitions

| **Research type** | **Definition** |
| --- | --- |
| Disciplinary (a.k.a. unidisciplinary, intradisciplinary) | ‘take[s] place within the bounds of a single, currently recognized academic discipline. (…) The research activity is orientated towards one specific goal, looking for an answer to a specific research question’ (Tress et al., 2006, p. 15). |
| Multidisciplinary | ‘the sequential or additive *combination* of ideas or methods drawn from two or more disciplines or fields to address a problem’ (Stokols et al., 2013, p. 5). |
| Interdisciplinary | ‘the *integration* of perspectives, concepts, theories, and methods from two or more disciplines or fields to address a problem’ (ibid). |
| Transdisciplinary | ‘an integrative process whereby scholars and practitioners from both academic disciplines and nonacademic fields work jointly to develop and use novel conceptual and methodological approaches that synthesize and extend discipline-specific perspectives, theories, methods, and translational strategies to yield innovative solutions to particular scientific and societal problems’ (ibid, p.6). |

Table 2 Summary of Stokols et al.’s (2013, p.9) four phases of transdisciplinary research

| **Research phase** | **Summary of goals (G) and processes (P) for each phase** |
| --- | --- |
| Development | Identify project and related fields/disciplines (G)  Form diverse team (G)  Develop shared understanding of problem and team mission (P)  Learn relevance of team members’ expertise and develop a shared vocabulary (P) |
| Conceptualisation | Develop research questions, hypotheses, conceptual framework and methods that ‘integrate and extend approaches’ across the team (G)  Use the process and ‘institutional environment to promote innovation and integration’ (P) |
| Implementation | Do the research (G)  Create refinements as needed ‘to maximize the effectiveness and innovative nature of the initiative.’ (G)  Be reflective to ‘assess and enhance activities, retaining an integrative approach’ (P) |
| Translation | Apply findings to develop solutions (G)  Support long-term participation of all partners (P) |

# Methods

We conducted a literature review using OVID Medline (27^th^ May 2019) exploring articles that conducted transdisciplinary research. Search terms for identifying transdisciplinary research studies were as follows: *1.transdiscip*.tw.; 2. Trans-discip*.tw.; 3. 1 or 2; 4. Research.tw.; 5. stud*.tw.; 6. 4 or 5; 7. health*.tw.; 8. 3 and 6 and 7.* There were no date restrictions. Papers were included if they provided substantive information about the methodology of the transdisciplinary research undertaken. We extracted data on the following: authors, year, country, study aims, methods, disciplines and non-academic stakeholders involved, and nature of conceptual framework.

We conducted a narrative synthesis using thematic analysis, coding studies inductively and deductively at the semantic level (Braun and Clarke, 2006; Fereday and Muir-Cochrane, 2006; Popay et al., 2006). We developed an *a priori* codebook for deductive coding using Stokols et al.’s (2013) transdisciplinary research framework (Table 2), using their four stages as coding categories (development, conceptualisation, implementation and translation) and we created codes within these using their goals and processes. We used inductive coding (involving the addition of new categories and codes) when data did not fit the *a priori* codebook. Data were analyzed using NVivo qualitative data analysis software (QSR International Pty Ltd., version 12.5.0, 2019). RWA coded all of the studies and HP coded a randomly selected portion (15%) and differences were discussed. RWA and HP collaboratively conducted the narrative synthesis, which involved re-reading coded data and iteratively developing preliminary models with additional concepts explored through tabulation and theme development (e.g. transdisciplinary challenges, strengths and opportunities).

We gathered feedback from experts in transdisciplinary research by contacting the included study authors using email and an online Google form (RWA) and by conducting a participatory workshop with 27 participants (HP, MD). The workshop was held at the 16^th^ International Conference on Urban Health in Xiamen, China on 4 November 2019 at a pre-conference session co-organised by the Wellcome Trust and their funded research projects under the Our Planet Our Health (OPOH) programme. Our approach was approved by a low risk departmental ethics approval, and participants gave consent. Our session lasted 75 minutes and involved a presentation of the preliminary model (HP) (see Figures 1 and 2) and presentations from three OPOH research projects relating their project to our preliminary model, including the Complex Systems for Sustainability and Health project (see ‘Reflections from the field’ in the main manuscript). The workshop presentation provided Stokols et al.’s (2013) definition and model of transdisciplinary research before introducing the research methods and preliminary model. This was followed by individual (10 minutes) and small group reflection (30 minutes) of four questions (see below) about participants’ experiences of transdisciplinary research, and similarities and differences with our model. The data from the workshop were analysed by reading and collating the responses in a single document, pulling out common reflections and illustrative examples (HP). A second researcher (RWA) read the feedback and potential changes to the model were discussed.

## Workshop feedback form questions

The following questions were posed at the participatory workshop on feedback forms:

1. Do you apply transdisciplinary research methods in your work? Why or why not?
2. How is this preliminary model similar of different to your transdisciplinary research experience?
3. Is this model missing any key processes that should be reflected?
4. Do you have any project examples or papers that show useful transdisciplinary research processes related to health and its wider determinants?

## Preliminary model

Figure 1 shows the preliminary version of the model in a simplified format and Figure 2 shows the detailed version.

Figure 1 Simple version of preliminary transdisciplinary research model

Figure 2 Detailed version of preliminary transdisciplinary research model

This second iteration of the model was sent to the included study authors for feedback. Study authors with published email addresses (n=41; note: this is more than the number of studies as some papers included email addresses for more than one author) were contacted via email twice in a four-week period and 4 responded. They were invited to review extracted data for their study (see Table 3) and provide feedback on the preliminary model. Comments were gathered via an online questionnaire (similar to the workshop feedback form, see below). As above, responses were used to complete Table 3 and to adapt the model (HP) and changes were discussed with the wider team.

## Online questionnaire questions

Authors of included studies were sent a link to the following online questionnaire. The instructions stated: ‘Using the text box below, please make comments, edits or corrections about your study in relation to how we've described it in the draft table of included studies and provide feedback on our preliminary model.

The questions stated:

1. Comments/feedback/corrections on the extracted data from your study (open text response)
2. First author of the paper you are commenting on (select one from list)
3. How is this preliminary model similar or different to your transdisciplinary research experience? (open text response)
4. Is this model missing any key processes that should be reflected? (open text response)
5. Do you have any other feedback on this model? (open text response)

# Results

## Description of included studies

Our search provided 529 results and following screening, 29 studies met our inclusion criteria (see Figure 3 for flow of results). The included studies (Table 3) were conducted internationally between 2002 and 2019. The included studies aimed to study and/or directly influence a diverse range of health conditions and wider determinants of health including: communicable and non-communicable diseases; risk factors such as climate change, sanitation infrastructure, and toxins; and health systems such as cardiac surgical care. The research methods varied considerably and included: literature review, participatory and action research methods, interviews and focus groups, artistic methods, statistical analysis and economic valuation. A majority of studies (76%, 22/29) partnered with non-academic stakeholders including: indigenous and other local populations, non-governmental organisations, multiple tiers of government, educators, farmers, and others. The scholarly/professional disciplines involved in the research were reported by 22 studies (including one via our online questionnaire).

Figure 3 Flow of results in review of studies conducting transdisciplinary research

Table 3 Description of transdisciplinary research methods in included papers. *Text or corrections provided by study authors via the online questionnaire.

| **Authors (Year)** | **Country** | **Study aim** | **Methods** | **Non-academic stakeholders** | **Disciplines/fields involved** |
| --- | --- | --- | --- | --- | --- |
| Anticona et al. (2013) | Peru | To identify environmental sources, pathways, and factors associated with lead and cadmium exposure among indigenous communities of the Peruvian Amazon. | Based upon the Ecohealth framework the research involved participatory design (organization of the partnership, agreement on the research question, principles, methods), and knowledge development (data collection, generation, dissemination of findings). Data collection involved sampling the population to determine lead and cadmium levels. Risk factors for exposure to lead and cadmium were assessed using a questionnaire. | Indigenous leaders, Pluspetrol (an oil and gas company in Latin America) and NGOs were involved in the research project design and conduct. | Epidemiology. |
| Archibald et al. (2018) | Canada | To develop an innovative knowledge translation tool based on rigorous research evidence and tailored to parents’ asthma-related information needs. | Literature review, qualitative study and arts-based knowledge translation tool development stage. | An artist, a creative writer, an illustrator, a digital media company. | Nursing, paediatrics, emergency medicine, and knowledge translation science. |
| Arenas-Monreal et al. (2015) | Mexico | To analyse the characteristics of health diagnosis, broadly encompassing morbidity and mortality, according to the Ecohealth approach in rural and urban communities in Mexico. | Mixed methodology with quantitative and qualitative components. | Local authorities, school teachers, religious representatives and popular groups (farmers, women), students and their families. | Medicine, nursing, epidemiology,  environmental engineering, anthropology, human nutrition  psychology, biochemistry, education. |
| Berger-González et al. (2016) | Guatemala | To create a tool for reciprocal reflexivity in multi-epistemological settings to study cancer healing systems of indigenous Mayan medical specialists from Guatemala and Western biomedical physicians. | Intra-group analysis (Emics of Self); Intra-group exchange (Etics of Other); Inter-group exchange; Intra-group exchange (Etics of Self); Inter-group exchange (Joint Etics). | The Maya partnership in this study comprised representatives from five ethnolinguistic councils. | Oncology, biology, medical anthropology, ethnopharmacology, physics, immunology, psychology, epidemiology, public health, environmental sciences. |
| Black and Black (2009) | Australia | To review the urban development and transport impacts on public health with particular reference to Australia. | Creation of a scientific team and conceptual framework, performing a literature review. | Stakeholder forums were used to identify research gaps and  formulate a draft set of research priorities. | Civil engineering, human geography, mathematics and statistics, mechanical engineering, education, environmental noise, evidence-based medicine, transport engineering, urban and regional planning. |
| Black et al. (2019) | UK | To understand how human and planetary health could become a priority for those who control the urban development process. | A quantitative economic valuation component and exploration and a qualitative action-research based exploration of barriers and opportunities for change. | The project team was led by individuals with real world experience in public health, urban development, and corporate decision-making. | Public health, economics, urban development. |
| Chastin et al. (2016) | Australia, Belgium, Brazil, Finland, France, Germany, Hong Kong, Ireland,  Italy,  Netherlands,  Norway,  UK  USA | To foster transdisciplinary system thinking; facilitate the identification of factors and cluster of factors influencing sedentary behaviour; guide secondary analyses of existing data; and prioritise research and guide targeted interventions and policy. | Concept mapping using a standardised mixed method, which combines qualitative opinions with multivariate statistical analysis to enable a group to gather and organise ideas into a conceptual framework. | Policy makers and practitioners were not involved and the transdisciplinary framework generated was based on scientific eminence and evidence. | Ageing science, anthropology, behavioural sciences, bioengineering, biostatistics, clinical biochemistry, clinical sciences.  developmental psychology, economics, epidemiology,  ergonomics, gerontology, health promotion, life sciences, measurement science, medicine, movement science, nutrition, paediatrics, physiotherapy, psychology, public health, rehabilitation sciences, social sciences, sport and exercise science, sports psychology, statistics, translational research. |
| Davies et al. (2015) | Australia, New Zealand | To describe a biogeographically dependent variation in airborne pollen diversity; a latitudinal gradient in the timing, duration and number of peaks of the grass pollen season; and the emergence of new methodologies based on transdisciplinary synthesis of aerobiology and remote sensing data. | A working group was established to systematically source, collate and analyse atmospheric pollen concentration data from11 Australian and six New Zealand sites. | Not known. | Botany, palynology, biogeography,  climate change science, plant genetics, biostatistics, ecology, pollen allergy, public health,  environmental health, medicine. |
| Dennis et al. (2009) | USA | To detail the application and utility of participatory photo mapping for studying the implications of place for the health of children. | Participatory photo mapping which combines participatory photography, community mapping, and lived experience interviews in order to capture both the qualitative and quantitative dimensions of people’s experience of place and health. | Images, narratives and other qualitative data were produced by participating community residents. | Landscape architecture, family medicine, population health sciences. |
| Fam and Sofoulis (2017) | USA | To design remote area water and sanitation systems in consultation with two native Alaskan communities. | Community engagement, analysis of social research data. | Native Alaskan community members were involved in the  sanitation designs. | Design, community engagement,  arctic engineers, civil engineers,  public health. |
| Gibson et al. (2002) | Canada | To develop a transdisciplinary model of health care priority setting. | Integration of an ethically-based account of health care priority setting with an empirically-based account to create a new transdisciplinary approach to the problem of priority setting. | Not known. | Not known. |
| Harper et al. (2012) | Canada | To gather data describing local impacts of climate change on health using community-directed digital storytelling. | Community participation, digital storytelling, in-depth interviews and a population survey. | Community researchers, regional and provincial Government. | Epidemiology, social sciences, public health, environmental sciences, development studies, biology. |
| Harper et al. (2008) | USA | To reduce HIV incidence and prevalence in youth 12 to 24 years old by developing and implementing community mobilization and structural change efforts in 15 different communities in the mainland United States and Puerto Rico. | A mixed-methods approach based upon a theoretical framework, involving a quantitative survey and semi-structured interviews. | Key leaders in the local community or individuals from community-based organizations. | Adolescent medicine, paediatric physician, community psychology, applied psychology, public health, communications. |
| Herrmann et al. (2017) | France, Germany, Norway, Sweden | To investigate how information on health co-benefits influences household’s choices of climate change mitigation actions in urban households in France, Germany, Norway, and Sweden. | A mixed methods approach combining quantitative and qualitative research methodologies. | Private consulting agency for climate policy and research, and policy advisory board with local, regional and national stakeholders from politics and society.* | Public health, psychology, political science, sustainability,  environmental sciences, economics.* |
| Holmes et al. (2008) | USA | To examine the highly dimensional, complex nature of multilevel health disparities and their effects on health. | Multi-level research and analysis, annual survey and social network analysis. | Community stakeholders, including schools, healthcare institutions, and community organization were involved as collaborators. | Biological sciences, clinical sciences, media and communications, public health policy and planning, social sciences, behavioural sciences,  biomechanics/statistics. |
| Ingemann et al. (2018) | Greenland | To examine the constraining and enabling determinants of the implementation processes within and across levels and sectors of Greenland’s second public health program. | Qualitative methods including semi-structured interviews, observations, telephone interviews, document analysis, focus groups discussions and a workshop. | Non-academic stakeholders (e.g. Ministry of Health, community health workers, schools and sports clubs) were involved in the research, in the identification of interactions and relationships across stakeholders and in all six phases of data collection. | Not known. |
| Kouamé et al. (2014) | Côte d’Ivoire | To assess the disease burdens related to unsustainable urban waste management in order to develop interventions that could improve the population’s well-being. | Water quality and risk factor assessments and the Ecohealth approach to analyse environmental and health threats. This involved a participatory workshop, mapping of environmental risk factors, cross-sectional household surveys and water sampling and testing. | Non-governmental organisations, farming communities, and the general population attended a participatory workshop. | Not known. |
| Lytle (2009) | USA | To develop effective innovative approaches with broad population impact at the social-environmental and policy levels for prevention of obesity, with particular emphasis on children and critical time periods during childhood where weight gain is likely. | A longitudinal cohort study. | Not known. | Urban planning, exercise physiology, nutrition, epidemiology, physiology, psychology. |
| Mosimann et al. (2017) | Mali | To elaborate a mixed methods approach providing key epidemiological and sociocultural data in order to evaluate the effectiveness of a dog rabies mass vaccination campaign carried out in two local areas in Mali with a view towards optimization for future scale-up. | A mixed methods approach was used that included triangulation of findings, comparative effectiveness model and interpretation. | Not known. | Not known. |
| Mutero et al. (2004) | Kenya | To study the links between malaria and agriculture in Mwea Division, Kenya using an ecosystems approach. | A mixed design involving five phases, including: stakeholder consultation and existing area health care utilization data collection; participatory needs assessment at the village level; household and farmer surveys; entomological and parasitological evaluation; and results dissemination workshops. | Each stage of the research had participation by the Mwea community. | Public health, crop and livestock science, medical anthropology,  sociology, medical entomology, parasitology, biostatistics. |
| Nastasi et al. (2015) | India | To develop a dynamic culturally con- structed clinical practice model for HIV/STI prevention that is (a) informed by research; (b) culturally co-constructed by married men, medical practitioners, and social scientists; and (c) adaptable to individual intra-cultural variations, that is, adaptable for use by different practitioners across a variety of patients. | Mixed methods in-depth ethnography with ongoing partnerships and continuing research to modify the newly developed model. | Partnerships were developed with key stakeholders including practitioners and community members. | Anthropology, psychology,  public health. |
| Quintero et al. (2009) | Columbia | To understand dengue as a bio-anthroposocial event that takes into account the interaction between biological, epidemiological, social and cultural data. | A cross-sectional study and triangulation including meteorological, entomological and qualitative methods. | Not known. | Medicine, epidemiology, sociology, anthropology, entomology, psychology. |
| Ramey et al. (2015) | USA | To identify the rationale and novel features of the Preconception Stress and Resiliency Pathways model and reflect on how it supports discourse, innovation in practice and longitudinal research. | Community-based participatory research. | The community was recognised  formally at all stages and community strengths and resources were identified. | Not known. |
| SantoDomingo et al. (2016) | Colombia | To adapt an ecohealth calendar with two indigenous communities in Colombia to integrate local knowledge of of eco-bio-social factors for vector-borne disease prevention, surveillance, and control. | The ecohealth calendars were constructed upon the triangulation of the information obtained with qualitative and participatory tools, using a transdisciplinary approach and based upon fieldwork, analytical, and dissemination phases. | The ecohealth calendars were designed with active participation from local actors and members of the Departmental Secretariats of Health. | Entomology, epidemiology, public health. |
| Scribner et al. (2017) | USA | To develop an approach to explaining health disparities in terms of social determinants. | Geospatial methods were used and linked data were analysed using quantitative multilevel models. | Not known. | Not known. |
| Thompson et al. (2015) | USA | To identify and prioritize hazards in cardiac surgery and to develop a patient safety agenda for cardiac surgical care. | Networked surveillance study, focused literature review, retrospective analysis of cardiovascular events, prospective peer assessment involving survey assessments, structured interviews, direct observations, and contextual inquiries. | Society of Cardiovascular Anesthesiology took part in the stakeholder modified nominal group review, reclassification, and prioritization of hazard data process. | Organizational sociology,  organizational psychology,  applied social psychology,  clinical medicine, human factors engineering, epidemiology, psychology, biostatistics, informatics, health services researchers. |
| Trujillo and Plough (2016) | USA | To create a “Making Health a Shared Value Action Area” this involves a set of national measures intended to drive fundamental improvement in opportunities to be healthy and stay healthy that are valued and accessible to everyone across the entire society. | Literature review, Stakeholder engagement, concept mapping, in-depth interviews and focus groups, and action framework and measures development. | Stakeholder engagement was undertaken in group concept mapping and in-depth interviews and focus groups exercises. | Not known. |
| Wade et al. (2010) | USA | To develop and implement a set of criteria for selecting health conditions and candidate genetic variants that could be included in a research prototype genetic susceptibility test. | Developing of selection criteria, genetic variant selection process including evidence review and recommendations. | The authors of this paper were from the National Institute of Health (USA), although they consider themselves to be “academics" (at least for the purposes of this paper) even though their site of employment is "government".* | Public health, sociology  behavioural research, bioethics,  genetic epidemiology. |
| Ziegler et al. (2016) | Thailand | To train students in ﬁeld research and gleaning interdisciplinary insights into persistent of *Opisthorchis viverrini* infection in northern Thailand and elucidating problems of focusing solely on the parasite as a means of addressing high prevalence of cholangiocarcinoma. | Qualitative fieldwork and a transdisciplinary design that created a dynamic, ever-evolving research process that demanded ﬂexibility, self-reﬂection, pedagogical sensitivity and cooperation. | The research team involved representatives from the Thai Ministry of Public Health. | Parasitology, pathology, epidemiology, surgery  ecology, hydrology, community healthcare, history, ethnography  geopolitics. |

## Description of feedback and changes to the model

Many participants at our workshop in Xiamen identified themselves as experienced in transdisciplinary research with 78% (21/27) having used such approaches. Those who reported that our preliminary model was similar to their work comprised 14 people (56%, 15/27), while five (19%, 5/27) wrote that it was different, and the remaining seven (26%, 7/27) either did not respond or gave an unclear response. Participants who agreed with the model described specific areas they liked, such as ‘*I especially agree with how the co-learning and reflection & refinement is happening continuously as the research is ongoing*.’ Another wrote ‘*Maybe I think it is better than my real experience*.’ Those who found the model different did not necessarily disagree with the model, but perhaps they identified gaps between their experience and the model. For example, one participant wrote ‘*Your model is more detailed in the process of establishing transdisciplinary research team, which is good for us to learn*.’ Differences (or missing components in our preliminary model) identified by participants included: leadership, data sharing for investigation and evaluation, a clearer process of evaluation, resource sharing across partners (see Table 4).

Questionnaire respondents provided comments supporting the model with two specific suggestions for alterations. Specific suggestions of missing components or areas to highlight included: finding a common language and development of cross-disciplinary awareness and respect. These were both added to the final model. In this article we have differentiated study quotes from workshop participants and questionnaire respondents using italics.

Table 4 Summary of feedback received from the participatory workshop and what changes were made as a result, if any. If no changes were made, this is justified.

| **Summarised comments from participatory workshop regarding missing components** | **Description of how comments were changed/not changed in the model** |
| --- | --- |
| A stage of development (e.g. for interventions) prior to implementation | Added text to the ‘Investigation’ stage: ‘Develop and test interventions’ but we have not pulled this out as a separate stage to avoid over-complicating the model. |
| Experimentation | Have not added because this is an implicit element of much research and the iterative nature of transdisciplinary research is already represented (such as through developing and testing interventions, as described above.) |
| Shared sense of ownership and sense of a mission | Added to ‘(Pre-)Development’ and ‘Co-learning’ |
| Leadership | Added the term ‘leadership’ in the detailed model. |
| Advocacy | Added to the model in ‘(Pre-)Development and ‘Implementation’ |
| querying whether investigators can be involved in implementation | Described in narrative of paper. |
| Key initiators required in the co-learning phase | Did not add because this level of detail may overly complicate the model and it could be argued that this is necessary for many activities. |
| Data sharing for investigation and evaluation | Added ‘data’ to ‘Reflection & Refinement’. |
| A clearer process of evaluation | Added to ‘Reflection & Refinement’. |
| Resource sharing across partners | Added to ‘Investigation’. |
| Additional feedback loops than those perceived in the model | Iterations of the model were drawn with arrows interconnecting all components. However this was visually very ‘messy’ and therefore we decided to encompass all stages in one large feedback loop to show that each stage is interconnected and that the process is iterative. |
| Questioning whether there are linear parts of transdisciplinary research. | Removed arrows between stages so that all stages are now connected in one large circular loop, which allows for some linear and many non-linear processes to occur. |
| Recognition of confusion and conflict | This is addressed in the ‘Requirements’ for the ‘Co-learning’ section: ‘Consensus over value of diverse knowledge types, Communication, Maintain trust & relationships’. |
| Possibility of receiving input on problem formulation from a very representative base of stakeholders | This is addressed by connecting the ‘Co-learning’ stage (which involves very broad engagement beyond the project) with the ‘(Pre-)Development stage’. |
| Querying why referring to EcoHealth and not Planetary Health | Removed specific methods and approaches as these were just examples. |
| Artistic methodologies added to multiple stages | Did not add to the model because specific methodologies are not included, however this is described narratively in the paper. |
| Power relations | Added to ‘Reflection & Refinement’ stage. |
| Querying addition of ‘singular or multiple’ approaches | Did not add because this level of detail may overly complicate the model. |
| Communication | Added to ‘Co-learning’ and ‘Reflection & Refinement’ stages |
| Add ‘share existing knowledge’ next to ‘create new knowledge’ | Added in ‘Co-learning’ stage. |
| Adding ‘study subjects’ to the first goal | Refined wording in ‘Co-learning’ to include widest possible group. |
| Potential additional activities about making impact by mapping responsibilities for change at global levels (e.g. for Planetary Health) and negotiating the ambitious vision of the research. | Added new ‘Requirements’ within each section and especially the ‘Implementation’ section regarding necessary factors for success. For this comment, we have added ‘Funding; Political or other stakeholder support; Other social, cultural, economic environmental factors; Advocacy’. |

## Simplified version of the model

Figure 4 shows a simplified version of the model for the purposes of communicating the overall process and relations between stages with wide stakeholders.

Figure 4 Simplified version of our new model of transdisciplinary research

## Comparison with Stokols et al.’s framework

In comparison with Stokols et al.’s (2013) framework, we have made six key changes. We removed the ‘Translation’ phase which was regularly described as an ongoing process, now represented within the cross-cutting ‘Co-learning’ stage. We added the ‘Investigation’ stage in which research is conducted and re-purposed Stokols et al.’s ‘Implementation’ stage so that it no longer refers to doing research and is now about making change happen beyond the research process. We added the ‘Reflection & Refinement’ stage as a cross-cutting process that recognises the need for ongoing evaluation and iteration over time. We have drawn out the pre-development activities in Stokols and colleagues’ ‘Development’ stage to demonstrate the wide range of time-consuming activities that occur prior to official project initiation. Finally, we modified the flow of the diagram from a cycle to include linear and cyclical processes that may feedback to project development, but not necessarily. The following sections present each stage of the new model and describe: an overall summary of the stage, examples from the literature/feedback to illustrate the activities in the stage, and a summary of challenges or requirements for success.

## Examples of implementation activities

Table 5 describes examples of ‘Implementation’ stage activities in transdisciplinary health research taken from the included studies.

Table 5 Examples of ‘Implementation’ stage activities in transdisciplinary health research

| **Implementation activity** | **Examples from included studies** |
| --- | --- |
| **Internal** stakeholders used results to improve health or the wider determinants of health | - Twelve design recommendations were produced for water and sanitation systems that would meet native communities’ needs in remote Alaska (Fam and Sofoulis, 2017). Engineers on the project used these results to design infrastructure systems. Each recommendation was accompanied by quotes and stories from residents which helped ‘helped to justify and validate the ﬁnal selection of system components within the project’ (p.1075) and engineers reported that they were a ‘valuable tool’ (p.1076). - Results from Thompson et al. (2015) were used to prioritise interventions to improve patient safety in cardiovascular surgery services from which they ‘developed tools specific to cardiac surgery and [they] are currently implementing them in a multi-institutional intervention’ (p.149). |
| **External** stakeholders used results to improve health or the wider determinants of health | - Young people and academic researchers co-presented participatory photo mapping results to community stakeholders, causing the following outcomes: ‘changes in the way community police ofﬁcers interact with neighborhood youth, youth-led neighborhood tours for local clinic staff, and physical improvements to neighborhood parks and pedestrian infrastructure’ (Dennis et al., 2009, p. 469). - Research results were presented to residents and local/municipal authorities, from which ‘collective decisions were taken to develop community initiatives to address some of the problems identified in the health diagnosis’ (Arenas-Monreal et al., 2015, p. 4). |
| Project developed, ran and evaluated an intervention | - Nastasi et al. (2015) worked with local partners to develop and run a local model for clinical practice about sexual health in low-income communities in Mumbai, first for men. They evaluated and iteratively improved the model. Then they developed capacity building programmes to ensure its sustainability in local practice. Finally they used the lessons learned to iteratively develop a further clinical model for women and couples. |
| Project partners sought additional funding, cycling back to ‘(Pre-) Development’ stage | - Ramey et al. (2015) developed a conceptual framework on the preconception period and its impact on parental and child health which they used to apply for funding for a prospective study on the topic. They reported discovering ‘how helpful this integrated framework was in identifying what to measure and the gaps in previous research’ and in supporting the network to using a shared vocabulary and stay ‘focused on understanding the interdependent, co-occurring, and sequential forces that likely impact health outcomes’ (p.e5). |
| Partners used the research outputs for advocacy, education or increasing research in an underrepresented topic | - The Robert Wood Johnson Foundation funded a transdisciplinary project that developed a ‘Culture of Health’ action framework with associated measures. This underpinned a new research programme at the philanthropy that recognises the impact of the social environment on health and health-related behaviours (Trujillo and Plough, 2016). - Archibald and colleagues (2018) used artistic methodologies, specifically storytelling and visual art, to provide educational material to parents of children with Asthma. |

# Recommended reporting of transdisciplinary research

We recommend that studies report the process-related factors in our recommended reporting criteria (Table 6), as appropriate for the individual study. This need not be a tick-box reporting exercise, but instead we aim for this to serve as a useful catalyst for reflection during the project from which others can learn.

Table 6 Recommended reporting criteria for transdisciplinary research about health and its wider determinants

| **Stage** | **Criteria** |
| --- | --- |
| (Pre-)Development | - How was the project team formed? - Which organisations are included and what are their roles? - How did partners agree on the problem or mission? |
| Co-learning | - Who attended project meetings? - What activities were used to create or share knowledge with diverse stakeholders? - If relevant, how were conflicts resolved about the value of diverse knowledge types (e.g. technical knowledge, experiential knowledge, etc.)? - How did the project build capacity within and beyond project partners? - How did partners build and maintain trust throughout the project? |
| Reflection & Refinement | - Which processes (or activities) were used to reflect upon the research and emerging results? - Which indicators were used to monitor progress/impact? - Who managed/participated in monitoring and reflection processes? |
| Conceptualisation | - How were diverse assumptions, theories and knowledge types integrated to form the project’s research questions and approach? - Which research governance processes were established and were these adjusted over time? - Did the project create/adopt a conceptual framework and/or Theory of Change and how did this occur? - Which activities were used to exchange perspectives and knowledge when conceptualising the research approach and who was involved? |
| Investigation | - Which methods were used to gather and analyse data and how were these methods novel or integrative? - How did the ‘Investigation’ stage build capacity within and beyond project partners? - Who was involved in gathering and analysing data and what were their respective roles? |
| Implementation | - How was (new) knowledge from this project used to solve problems and or improve health? Or how do partners anticipate such change occurring? - Which factors were required to make this change happen (e.g. funding or political will) and how were these achieved? |

# References

Anticona, C., Coe, A.-B., Bergdahl, I.A., San Sebastian, M., 2013. Easier said than done: challenges of applying the Ecohealth approach to the study on heavy metals exposure among indigenous communities of the Peruvian Amazon. BMC Public Health 13, 437. https://doi.org/10.1186/1471-2458-13-437

Archibald, M.M., Hartling, L., Ali, S., Caine, V., Scott, S.D., 2018. Developing “My Asthma Diary”: a process exemplar of a patient-driven arts-based knowledge translation tool. BMC Pediatr. 18, 186. https://doi.org/10.1186/s12887-018-1155-2

Arenas-Monreal, L., Cortez-Lugo, M., Parada-Toro, I., Pacheco-Magaña, L.E., Magaña-Valladares, L., 2015. Population health diagnosis with an ecohealth approach. Rev. Saúde Pública 49. https://doi.org/10.1590/S0034-8910.2015049005842

Berger-González, M., Stauffacher, M., Zinsstag, J., Edwards, P., Krütli, P., 2016. Transdisciplinary Research on Cancer-Healing Systems Between Biomedicine and the Maya of Guatemala: A Tool for Reciprocal Reflexivity in a Multi-Epistemological Setting. Qual. Health Res. 26, 77–91. https://doi.org/10.1177/1049732315617478

Black, D., Black, J., 2009. A review of the urban development and transport impacts on public health with particular reference to Australia: trans-disciplinary research teams and some research gaps. Int. J. Environ. Res. Public Health 6, 1557–1596. https://doi.org/10.3390/ijerph6051557

Black, D., Scally, G., Orme, J., Hunt, A., Pilkington, P., Lawrence, R., Ebi, K., 2019. Moving Health Upstream in Urban Development: Reflections on the Operationalization of a Transdisciplinary Case Study. Global Challenges 3, 1700103. https://doi.org/10.1002/gch2.201700103

Braun, V., Clarke, V., 2006. Using thematic analysis in psychology. Qualitative Research in Psychology 3, 77–101. https://doi.org/10.1191/1478088706qp063oa

Chastin, S.F.M., De Craemer, M., Lien, N., Bernaards, C., Buck, C., Oppert, J.-M., Nazare, J.-A., Lakerveld, J., O’Donoghue, G., Holdsworth, M., Owen, N., Brug, J., Cardon, G., DEDIPAC consortium, expert working group and consensus panel, 2016. The SOS-framework (Systems of Sedentary behaviours): an international transdisciplinary consensus framework for the study of determinants, research priorities and policy on sedentary behaviour across the life course: a DEDIPAC-study. Int. J. Behav. Nutr. Phys. Act. 13, 83. https://doi.org/10.1186/s12966-016-0409-3

Davies, J.M., Beggs, P.J., Medek, D.E., Newnham, R.M., Erbas, B., Thibaudon, M., Katelaris, C.H., Haberle, S.G., Newbigin, E.J., Huete, A.R., 2015. Trans-disciplinary research in synthesis of grass pollen aerobiology and its importance for respiratory health in Australasia. Sci. Total Environ. 534, 85–96. https://doi.org/10.1016/j.scitotenv.2015.04.001

Dennis, S.F., Gaulocher, S., Carpiano, R.M., Brown, D., 2009. Participatory photo mapping (PPM): Exploring an integrated method for health and place research with young people. Health & Place 15, 466–473. https://doi.org/10.1016/j.healthplace.2008.08.004

Fam, D., Sofoulis, Z., 2017. A “Knowledge Ecologies” Analysis of Co-designing Water and Sanitation Services in Alaska. Sci. Eng. Ethics 23, 1059–1083. https://doi.org/10.1007/s11948-016-9830-x

Fereday, J., Muir-Cochrane, E., 2006. Demonstrating Rigor Using Thematic Analysis: A Hybrid Approach of Inductive and Deductive Coding and Theme Development. International Journal of Qualitative Methods 5, 80–92. https://doi.org/10.1177/160940690600500107

Gibson, J.L., Martin, D.K., Singer, P.A., 2002. Priority setting for new technologies in medicine: a transdisciplinary study. BMC Health Serv. Res. 2, 14.

Harper, G.W., Neubauer, L.C., Bangi, A.K., Francisco, V.T., 2008. Transdisciplinary research and evaluation for community health initiatives. Health Promot. Pract. 9, 328–337. https://doi.org/10.1177/1524839908325334

Harper, S.L., Edge, V.L., Cunsolo Willox, A., Rigolet Inuit Community Government, 2012. “Changing climate, changing health, changing stories” profile: using an EcoHealth approach to explore impacts of climate change on inuit health. Ecohealth 9, 89–101. https://doi.org/10.1007/s10393-012-0762-x

Herrmann, A., Fischer, H., Amelung, D., Litvine, D., Aall, C., Andersson, C., Baltruszewicz, M., Barbier, C., Bruyère, S., Bénévise, F., Dubois, G., Louis, V.R., Nilsson, M., Richardsen Moberg, K., Sköld, B., Sauerborn, R., 2017. Household preferences for reducing greenhouse gas emissions in four European high-income countries: Does health information matter? A mixed-methods study protocol. BMC Public Health 18, 71. https://doi.org/10.1186/s12889-017-4604-1

Holmes, J.H., Lehman, A., Hade, E., Ferketich, A.K., Gehlert, S., Rauscher, G.H., Abrams, J., Bird, C.E., 2008. Challenges for multilevel health disparities research in a transdisciplinary environment. Am. J. Prev. Med. 35, S182-92. https://doi.org/10.1016/j.amepre.2008.05.019

Ingemann, C., Regeer, B.J., Larsen, C.V.L., 2018. Determinants of an integrated public health approach: the implementation process of Greenland’s second public health program. BMC Public Health 18, 1353. https://doi.org/10.1186/s12889-018-6253-4

Kouamé, P.K., Dongo, K., Nguyen-Viet, H., Zurbrügg, C., Lüthi, C., Hattendorf, J., Utzinger, J., Biémi, J., Bonfoh, B., 2014. Ecohealth approach to urban waste management: exposure to environmental pollutants and health risks in Yamoussoukro, Côte d’Ivoire. Int. J. Environ. Res. Public Health 11, 10292–10309. https://doi.org/10.3390/ijerph111010292

Lytle, L.A., 2009. Examining the etiology of childhood obesity: The IDEA study. Am. J. Community Psychol. 44, 338–349. https://doi.org/10.1007/s10464-009-9269-1

Mosimann, L., Traoré, A., Mauti, S., Léchenne, M., Obrist, B., Véron, R., Hattendorf, J., Zinsstag, J., 2017. A mixed methods approach to assess animal vaccination programmes: The case of rabies control in Bamako, Mali. Acta Trop. 165, 203–215. https://doi.org/10.1016/j.actatropica.2016.10.007

Mutero, C.M., Kabutha, C., Kimani, V., Kabuage, L., Gitau, G., Ssennyonga, J., Githure, J., Muthami, L., Kaida, A., Musyoka, L., Kiarie, E., Oganda, M., 2004. A transdisciplinary perspective on the links between malaria and agroecosystems in Kenya. Acta Trop. 89, 171–186. https://doi.org/10.1016/j.actatropica.2003.07.003

Nastasi, B.K., Schensul, J.J., Schensul, S.L., Mekki-Berrada, A., Pelto, P.J., Maitra, S., Verma, R., Saggurti, N., 2015. A model for translating ethnography and theory into culturally constructed clinical practices. Cult. Med. Psychiatry 39, 92–120. https://doi.org/10.1007/s11013-014-9404-9

Popay, J., Roberts, H., Sowden, A., Petticrew, M., Arai, L., Rodgers, M., Britten, N., Roen, K., Duffy, S., 2006. Guidance on the conduct of narrative synthesis in systematic reviews. A product from the ESRC methods programme Version 1, b92.

Quintero, J., Carrasquilla, G., Suárez, R., González, C., Olano, V.A., 2009. An ecosystemic approach to evaluating ecological, socioeconomic and group dynamics affecting the prevalence of Aedes aegypti in two Colombian towns. Cad. Saúde Pública 25, s93–s103. https://doi.org/10.1590/S0102-311X2009001300009

Ramey, S.L., Schafer, P., DeClerque, J.L., Lanzi, R.G., Hobel, C., Shalowitz, M., Chinchilli, V., Raju, T.N.K., Community Child Health Network, 2015. The Preconception Stress and Resiliency Pathways Model: a multi-level framework on maternal, paternal, and child health disparities derived by community-based participatory research. Matern. Child Health J. 19, 707–719. https://doi.org/10.1007/s10995-014-1581-1

SantoDomingo, A.F., Castro-Díaz, L., González-Uribe, C., Wayúu Community of Marbacella and El Horno, Barí Community of Karikachaboquira, 2016. Ecosystem Research Experience with Two Indigenous Communities of Colombia: The Ecohealth Calendar as a Participatory and Innovative Methodological Tool. Ecohealth 13, 687–697. https://doi.org/10.1007/s10393-016-1165-1

Scribner, R.A., Simonsen, N.R., Leonardi, C., 2017. The Social Determinants of Health Core: Taking a Place-Based Approach. Am. J. Prev. Med. 52, S13–S19. https://doi.org/10.1016/j.amepre.2016.09.025

Stokols, D., Hall, K., Vogel, A., 2013. Transdisciplinary Public Health: Definitions, Core Characteristics, and Strategies for Success, in: Joshu, D., McBride, T.D. (Eds.), Transdisciplinary Public Health: Research, Methods, and Practice. Jossey-Bass Publishers, San Francisco, USA, pp. 3–30.

Thompson, D.A., Marsteller, J.A., Pronovost, P.J., Gurses, A., Lubomski, L.H., Goeschel, C.A., Gosbee, J.W., Wahr, J., Martinez, E.A., 2015. Locating Errors Through Networked Surveillance: A Multimethod Approach to Peer Assessment, Hazard Identification, and Prioritization of Patient Safety Efforts in Cardiac Surgery. J. Patient Saf. 11, 143–151. https://doi.org/10.1097/PTS.0000000000000059

Tress, B., Tress, G., Fry, G., 2006. Defining concepts and the process of knowledge production in integrative research, in: Tress, B., Tress, G., Fry, G., Opdam, P. (Eds.), From Landscape Research to Landscape Planning: Aspects of Integration, Education and Application, Wageningen UR Frontis Series. Springer, Dordrecht, pp. 13–26.

Trujillo, M.D., Plough, A., 2016. Building a culture of health: A new framework and measures for health and health care in America. Soc. Sci. Med. 165, 206–213. https://doi.org/10.1016/j.socscimed.2016.06.043

Wade, C.H., McBride, C.M., Kardia, S.L.R., Brody, L.C., 2010. Considerations for designing a prototype genetic test for use in translational research. Public Health Genomics 13, 155–165. https://doi.org/10.1159/000236061

Ziegler, A.D., Echaubard, P., Lee, Y.T., Chuah, C.J., Wilcox, B.A., Grundy-Warr, C., Sithithaworn, P., Petney, T.N., Laithevewat, L., Ong, X., Andrews, R.H., Ismail, T., Sripa, B., Khuntikeo, N., Poonpon, K., Tungtang, P., Tuamsuk, K., 2016. Untangling the Complexity of Liver Fluke Infection and Cholangiocarcinoma in NE Thailand Through Transdisciplinary Learning. Ecohealth 13, 316–327. https://doi.org/10.1007/s10393-015-1087-3
